# Smart grading: A generative AI-based tool for knowledge-grounded answer evaluation in educational assessments

**DOI:** 10.1016/j.mex.2023.102531

**Published:** 2023-12-20

**Authors:** Samuel Tobler

**Affiliations:** ETH Zurich, Switzerland

**Keywords:** GenAI-Based Smart Grading, Artificial intelligence, Test evaluation, Educational assessment, Automated grading, GPT, Large language model

## Abstract

Evaluating text-based answers obtained in educational settings or behavioral studies is time-consuming and resource-intensive. Applying novel artificial intelligence tools such as ChatGPT might support the process. Still, currently available implementations do not allow for automated and case-specific evaluations of large numbers of student answers. To counter this limitation, we developed a flexible software and user-friendly web application that enables researchers and educators to use cutting-edge artificial intelligence technologies by providing an interface that combines large language models with options to specify questions of interest, sample solutions, and evaluation instructions for automated answer scoring. We validated the method in an empirical study and found the software with expert ratings to have high reliability. Hence, the present software constitutes a valuable tool to facilitate and enhance text-based answer evaluation.

• Generative AI-enhanced software for customizable, case-specific, and automized grading of large amounts of text-based answers.
• Open-source software and web application for direct implementation and adaptation.

## Graphical abstract

**Figure:**
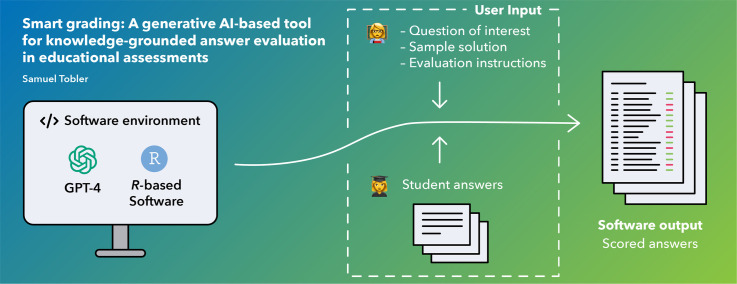

Specifications table

**Table:** 

Subject area:	Psychology
More specific subject area:	Educational Assessment
Name of your method:	GenAI-Based Smart Grading
Name and reference of original method:	NA
Resource availability:	Web application: www.stobler.shinyapps.io/smartgrading Code and software: www.github.com/samueltobler/smartgrading

## Method details

### Background

Evaluating coursework or answers to open questions, such as in educational environments or empirical behavioral studies, is tedious work that is cost and resource-intensive. Being able to case-specifically evaluate an answer's content and assign points accordingly, making use of generative artificial intelligence (GenAI) might present a solution for this problem. GenAI functions by integrating sophisticated natural language processing algorithms aiming to understand the context, extract the relevant information, and provide detailed responses. However, currently available large language model (LLM) applications, such as ChatGPT [14], Claude 2 [2], or Google's Bard [5], are limited regarding a) factual correctness or specificity to the expected solution (despite their recent developments, see [6]) and b) amount of data that can be provided and analyzed in an automatized and sequential way. As such, it is impossible to provide an extensive data set with student answers to be graded automatically and based on priorly defined instructions. Instead, researchers or educators would need to evaluate each answer individually by repetitively providing sample solutions, evaluation instruction, and point assignments when using GenAI tools, resulting in time-consuming efforts to integrate artificial intelligence in analyzing answers to open questions.

OpenAI's generative pre-trained transformer (GPT) developer framework enables the independent development of functions and applications using cutting-edge LLM technologies. Integrating these GenAI technologies, we present a method in which answers to open questions, their sample solution, and grading instructions are sufficient to evaluate large amounts of student answers automatically. Moreover, we apply this novel method to provide the code for research question-specific adaptations and easy-to-use software for directly applying the method without programming requirements.

### How the software works

The pseudocode depicted in the code box below shows the logic underlying the function and software. Specifically, the process makes use of the LLM's large context windows to specify questions, sample solutions, and evaluation guidelines integrated into the system's instruction prompt. As such, GenAI-based chat completion is applied to the specific use case.

The user's input corresponds to a student's answer to the question of interest, which is individually evaluated. The resulting evaluation score is then saved in a vector, which can be later exported for further data processing or analysis. Repeating this procedure for all participants’ answers allows obtaining the desired ratings, scores, or grades.

**Fig. 1:**
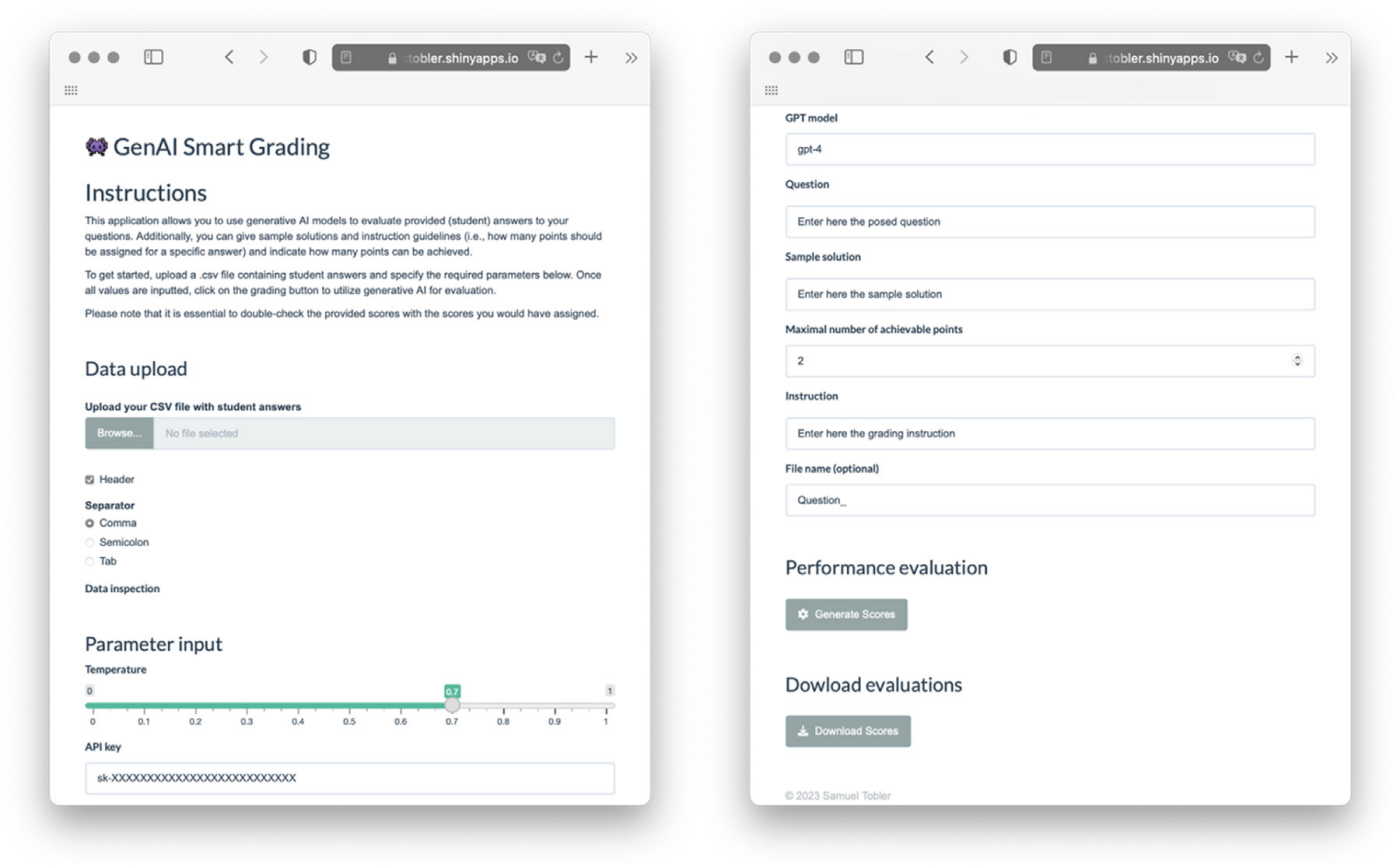
Screenshot of the web application. The web application can be used by uploading a “.csv”-file of the student answers. Upon successful upload, the first few rows of the data are displayed and can be inspected. This enables the user to adjust the input file concerning whether there is a header or which separator was used in the data file. Subsequently, the individual parameters must be specified, including the OpenAI API key, which must be generated in advance. After completing these steps, the performance can be evaluated by clicking the “Generate Scores” button, and the results can be downloaded as a “.csv” file. No data is stored when using the software. Alternatively, the software can be directly run from the *R-*software environment [16].

To run the application successfully, various parameters must be defined in advance. These include GPT-related parameters (OpenAI API key, temperature, and model) and test-related parameters (question, sample solution, evaluation instructions, points, and recorded answers), which are explained in Table 1. A screenshot of the web application software is shown in Fig. 1.

**Table 1:** Overview of the required parameters and variables.

Parameter or variable	Description
*GPT-related*	
OpenAI API Key	An OpenAI API key is essential to make use of their LLMs, also considering the associated computing costs. The API key can be generated on their website after creating a user account.
Temperature	The temperature parameter describes the model's randomness when generating an answer. Higher values (closer to 1) result in less predictable responses, while lower values (closer to 0) make the model's output more deterministic. We recommend adjusting the temperature parameters based on the specific requirements of the situation. If short answers are evaluated, a lower temperature and, thus, a higher deterministic output might be better suited. A less predictable output might be favorable for longer answers to allow for more diverse responses.
Model	We recommend gpt-4-based models such as gpt-4, gpt-4-0610, or more recent models for higher accuracy. Considering the current speed of development, it might quickly occur that the herein-described models are, at some point, outdated. However, the general structure of the application or function is easily adjustable for more advanced models that are yet to come. Accordingly, the application requires a manual model specification. It is also worth noticing that the prices of individual models differ, whereas, generally speaking, more recent models have higher token costs.
*Test-related*	
Question	Question or exercise that has been solved. It is essential to acknowledge that well-written and clearly stated questions result in higher accuracy when evaluating the answers. For instance, asking students to explain a concept might involve more possible solutions than reformulating it and asking for three differences between two related ideas.
Sample solution	The perfect solution for the question of interest. Similarly, specifying different answer options and writing a complete sample solution increases the evaluation accuracy, considering that the LLM will be asked to use this information as the basis for evaluation.
Evaluation instructions	Evaluation instructions can be used to specify when and for which aspects to give points, when to take points away, and which answer option counts how much. For instance, when asking for three differences, it might make sense to instruct the LLM to give one point per argument.
Points	Points indicate how many points might be assigned to individual answers.
Answers	The students’ answers must be provided as individual elements of a vector, for instance, by saving them as a single column «.csv» file.

Employing the current method to enhance one's research designs or facilitate data handling might be done in various ways. First, researchers might adapt the herein-described code to their purposes; second, researchers might use the provided functions as code to implement them in their data analysis pipelines; or third, researchers might run the provided software with adjustable default settings.

**Code box**. Pseudocode explanation of the method's primary function.

**Table:** 

**Structure of the software**	**Structure of the SmartGrading function**
Initialize	Initialize
Inputs	Inputs
OPENAI key	OPENAI key, GPT-model, model temperature, question, sample solution, evaluation instruction, points, answer
GPT-model	Outputs
Model temperature	Score
Question	Main
Sample solution	Call OpenAI Chat Completion Function
Evaluation instruction	Define inputs
Points	Define chat completion via JSON mode
Answers (to be evaluated)	Message
Outputs	Role: system
Scores	Content: “You have to give points to a user- generated answer to this question with the
Main	Following sample solution. Follow these evaluation instructions assigning maximally this number of points”
For all participants	Role: user
Run SmartGrading function	Content: answer
Store obtained score in Scores vector	Return Score
Return Scores vector	
Export Scores vector	

*Note.* The herein-described functions are either available as code on GitHub or directly applicable using the *GenAI Smart Grading* web application. Note that the actual system message of the *SmartGrading* function contains a more fine-grained elaboration of how the different inputs must be handled.

### When to use the software

The following section presents different potential use cases of the proposed method.

1. *Enhance formative assessments in educational settings.* Formative assessments, i.e., “[the] process used … to recognize and respond to student learning in order to enhance that learning” (Bell & Cowie, 2000, p. 1 [3]) have been repeatedly shown to increase students’ follow-up performance [9,12], potentially due to higher meta-cognitive calibration, knowledge gap awareness, or self-regulating strategies [4,13]. Accordingly, such assessments are often implemented in educational settings. However, many answers must be corrected if open questions are used in these non-graded assessments. To be able to a) frequently conduct such formative assessments (also for educators to assess the knowledge status of the class) or b) directly discuss the results in class without time-consuming correction efforts, the application can easily be used to grade all students’ answers with only few time investment. Hence, this approach allows teachers to incorporate teaching strategies directly to tackle frequent misconceptions and adjust teaching. Moreover, it might promote a shift from clicker-based in-class formative assessments to more informative open answers.
2. *Evaluate open answers to test items in behavioral studies.* While procedural and conceptual knowledge are often questioned with multiple-choice items, transfer tasks mainly involve open-ended questions due to their intrinsic thematic difficulty and the associated challenges to formulate multiple-choice questions accordingly [17]. To support data pre-processing for subsequent analysis, the herein-presented method might be used to reduce the researchers' correcting efforts. Moreover, this approach also allows non-domain experts to correct test items in case a comprehensive sample solution is available, such as suggested for open-ended questions in various assessment contexts (see Hofer et al. [8] for general guidelines). Therefore, having a sample solution alone often requires specific content knowledge to interpret and contextualize the collected answers. Hence, this tool might support this evaluation process by basing answers on a more extensive knowledge base outside the researcher's domain expertise.
3. *Double-check your grading of coursework.* Suppose handling large amounts of coursework to be corrected. In that case, it might be helpful to use the software to check one's evaluations double to counteract potential biases (e.g., tendencies to grade first and last answers better or individual biases when grading; see [7] or Protivínský & Münich [15], for instance). This becomes specifically important when anonymous grading is not possible. By obtaining a second less biased AI-based score, disagreements can be efficiently double-checked and, if required, adjusted accordingly.
4. *Adaptations to other purposes.* The instructions of the provided function or software might also easily be adjusted to implement a weighting of different answers to group and analyze them, for instance.

## Method validation

Data from an empirical behavioral study were used to evaluate the method's applicability and validity. The study investigated the effect of different instructional conditions on subsequent post-test performance in a randomized control trial. The post-test consisted of various items, of which the responses to one open question were chosen as sample data for the current validation experiment.

The study participants were undergraduate or post-graduate natural science or technology students from a highly ranked Swiss university (*n* = 29; Age (*Mean ± SD*): 23.9 *±* 3.4 years old; 58.6% female, 41.4% male, 0% non-binary). Their answers were manually evaluated and additionally graded by using the developed software as numerical scores. An example of two provided answers, the sample solution (i.e., scoring rubric), as well as the manual and the *SmartGrading*-based scores are displayed in Table 2.

**Table 2:** Exemplary student answers and their evaluation.

Question & Grading instruction	Sample solution	Student answer	Manually assigned Score	Smart-Grading Score
Name two aspects of how the surface level is different from the textbase level in Kintsch's Construction Integration (C-I) model. Each correct aspect gives one point. (Maximally 2 Points)	The surface Level involves decoding words and sentences, focuses on recognizing individual words, their syntax, and semantics, and the proficiency at this level is essential for comprehension. The textbase level involves constructing text-based propositions and their relationships, represents actions, events, or states of affairs extracted from the text and requires making inferences, filling gaps, and resolving ambiguities for coherent understanding.	A1: “it was just about understanding the language i think and the text based level is more about the deeper step of understanding the given text”	1	1
		A2: “ability to read / recognise words vs. ability to imagine situation”	2	2

**Fig. 2:**
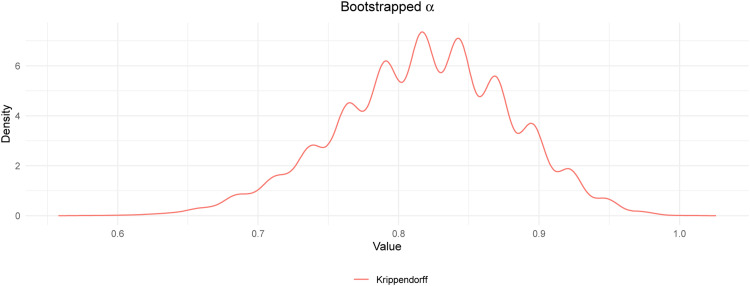
Density plot of bootstrapped Krippendorff's α.

To estimate the inter-rater reliability of the assigned points (i.e., the degree of agreement between the manual and the *SmartGrading*-based assigned scores), Krippendorff's α [11] was determined using the Krippendorff function of the *icr* package [18]. The 95% confidence interval was determined by bootstrapping of 10’000 samples. The results indicated high agreement between the manual and AI-based grading (α= 0.818, *SE* = 0.061, 95% CI [0.689, 0.926]; Fig. 2). Discrepancies mainly arose from a stricter evaluation by the software. Hence, the findings indicate that the developed approach represents a reliable tool to evaluate student answers if providing the question and a sample solution. An analysis script to conduct similar reliability estimations is available on GitHub.

## Limitations and recommendations

Whereas the present method and the resulting software entail various options to facilitate data analysis and scoring in classroom and empirical study settings, there are also limitations associated with this approach that can be grouped into GPT-associated, test-associated, and ethical limitations.

*GPT-associated limitations*: Whereas current models perform well in text recognition and generation tasks, there is currently no satisfactory solution for integrating figures or mathematical formulas. One way to counter these limitations is by meticulously describing figures or using LaTeX to depict mathematical content [10]. Moreover, artificial hallucinations may occur that lead to the generation of incorrect replies and answering patterns [1]. Yet, by precisely defining the system's instruction (see code box), these hallucinations can be countered to a certain extent.

*Test-associated challenges*: Complex or nested questions might be more challenging to evaluate since the algorithm could struggle to extract the relevant information and connections correctly. These difficulties appear to be even more pronounced if questions are not clearly formulated or the sample solution lacks some details. Similarly, providing detailed evaluation instructions is essential to assign points as intended. To double-check the grading, it might also be recommendable to determine Krippendorff's α of a subset of the original data. An *R*-script to do so is also available on GitHub, together with all other functions and software code.

*Ethical limitations*: Whereas this software could be used to grade students' exams, we strongly urge not to make students' passing or failing of an exam, course, or study dependent on an AI-based evaluation tool. Nonetheless, as discussed above, the software might be applied in various non-graded formats, such as formative assessments or to double-check on gradings. Another ethical consideration concerns the data safety of students. When using OpenAI's GPT models, the included data will be processed and potentially stored on external servers. Hence, students’ informed consent might be required. Yet, by providing answers to open questions without any identifying data, the student's personal information can be largely protected.

## Future work

Future work will center on advancing the present software to align with prospective technological advancements, especially in the realm of more accurate GPTs that produce less artificial hallucinations, display higher factual correctness, or can work with diverse answer inputs, including formulas, diagrams, or acoustic elements. Additionally, basing the provided grades not only on the predefined sample solution but also by incorporating specific information provided by the educator or researcher (such as instructional materials, lecture contents, or book chapters) might further enhance the accuracy of the grading.

## Conclusion

In conclusion, evaluating large amounts of text answers from educational assessments or behavioral studies is time and resource-intensive, thus slowing down educational interventions and progress in research. Here, we present and validate a method and software that implement generative AI to automate the scoring of extensive text-based answers according to provided sample solutions and grading instructions.

## Ethics statements

As part of the validation experiment, students' performance data in a non-graded, course-unrelated test was collected fully anonymized. All participants were informed about the study beforehand and gave informed consent before participating. The local university's ethics commission accepted the study before its conduct (EK-2023-N-212).

## CRediT authorship contribution statement

**Samuel Tobler:** Conceptualization, Methodology, Software, Validation, Formal analysis, Investigation, Resources, Data curation, Writing – original draft, Visualization, Project administration.

## Declaration of Competing Interest

The authors declare that they have no known competing financial interests or personal relationships that could have appeared to influence the work reported in this paper.

## Data Availability

The link to the software, codes, and analysis scripts has been shared. Data of the validation study upon request.
